# Dengue virus in humans and mosquitoes and their molecular characteristics in northeastern Thailand 2016-2018

**DOI:** 10.1371/journal.pone.0257460

**Published:** 2021-09-14

**Authors:** Patcharaporn Nonyong, Tipaya Ekalaksananan, Supranee Phanthanawiboon, Sirinart Aromseree, Juthamas Phadungsombat, Emi E. Nakayama, Tatsuo Shioda, Vorthon Sawaswong, Sunchai Payungporn, Kesorn Thaewnongiew, Hans J. Overgaard, Michael J. Bangs, Neal Alexander, Chamsai Pientong

**Affiliations:** 1 Department of Microbiology, Faculty of Medicine, Khon Kaen University, Khon Kaen, Thailand; 2 HPV & EBV and Carcinogenesis Research Group, Khon Kaen University, Khon Kaen, Thailand; 3 Mahidol-Osaka Center for Infectious Diseases, Faculty of Tropical Medicine, Mahidol University, Bangkok, Thailand; 4 Research Institute for Microbial Diseases, Osaka University, Osaka, Japan; 5 Program in Bioinformatics and Computational Biology, Graduate School, Chulalongkorn University, Bangkok, Thailand; 6 Department of Biochemistry, Faculty of Medicine, Chulalongkorn University, Bangkok, Thailand; 7 Department of Disease Control, Office of Disease Prevention and Control, Region 7 Khon Kaen, Ministry of Public Health, Khon Kaen, Thailand; 8 Faculty of Science and Technology, Norwegian University of Life Sciences, Ås, Norway; 9 Public Health & Malaria Control, PT Freeport Indonesia/International SOS, Kuala Kencana, Papua, Indonesia; 10 Department of Entomology, Faculty of Agriculture, Kasetsart University, Bangkok, Thailand; 11 MRC Tropical Epidemiology Group, London School of Hygiene and Tropical Medicine, London, United Kingdom; Defense Threat Reduction Agency, UNITED STATES

## Abstract

Dengue is hyperendemic in most Southeast Asian countries including Thailand, where all four dengue virus serotypes (DENV-1 to -4) have circulated over different periods and regions. Despite dengue cases being annually reported in all regions of Thailand, there is limited data on the relationship of epidemic DENV infection between humans and mosquitoes, and about the dynamics of DENV during outbreaks in the northeastern region. The present study was conducted in this region to investigate the molecular epidemiology of DENV and explore the relationships of DENV infection in humans and in mosquitoes during 2016–2018. A total of 292 dengue suspected patients from 11 hospitals and 902 individual mosquitoes (at patient’s houses and neighboring houses) were recruited and investigated for DENV serotypes infection using PCR. A total of 103 patients and 149 individual mosquitoes were DENV -positive. Among patients, the predominant DENV serotypes in 2016 and 2018 were DENV-4 (74%) and DENV-3 (53%) respectively, whereas in 2017, DENV-1, -3 and -4 had similar prevalence (38%). Additionally, only 19% of DENV infections in humans and mosquitoes at surrounding houses were serotypically matched, while 81% of infections were serotypically mismatched, suggesting that mosquitoes outside the residence may be an important factor of endemic dengue transmission. Phylogenetic analyses based on envelope gene sequences showed the genotype I of both DENV-1 and DENV-4, and co-circulation of the Cosmopolitan and Asian I genotypes of DENV-2. These strains were closely related to concurrent strains in other parts of Thailand and also similar to strains in previous epidemiological profiles in Thailand and elsewhere in Southeast Asia. These findings highlight genomic data of DENV in this region and suggest that people’s movement in urban environments may result in mosquitoes far away from the residential area being key determinants of DENV epidemic dynamics.

## Introduction

Dengue, caused by the dengue virus (DENV), is one of the most important re-emerging arboviral diseases in the tropical and subtropical regions of the world, including Southeast Asia. The virus is transmitted between humans primarily by the mosquitoes *Aedes aegypti*, and *Ae*. *albopictus* being a secondary vector [1]. The disease is endemic in more than 100 countries, with more than 2.5 billion people at risk [2]. There are an estimated 390 million DENV infections annually, of which 96 million are symptomatic, ranging from mild dengue fever, with or without warning signs, to severe dengue with plasma leakage that may lead to shock, bleeding, and/or organ impairment [3]. Increasing numbers of dengue cases, and the propagation of all four serotypes of DENV, are facilitated by uncontrolled urbanization, suboptimal management of water and solid waste, gaps in vector control, and rapid population movement, especially travel [4, 5]. In the absence of specific antiviral drugs and an effective vaccine, virus surveillance for early warning and integrated vector management are the primary options for the prevention and control of dengue outbreaks [6, 7]. Dengue prevention and control programs in Thailand are mainly based on hospital case reporting, conducted jointly by the hospital and the Offices of Disease Prevention and Control (ODPC), Ministry of Public Health. The dengue surveillance team responds when a case is reported by a hospital within 24 hours of notice in order to prevent transmission by spraying insecticides within 100 meters of the patient’s house [8].

DENV is a single-stranded, positive-sense RNA virus within the *Flaviviridae* family, and has four distinct serotypes: DENV-1 to -4 [9]. The viral genome has length approximately 11 kb which contains a single open reading frame (ORF) encoding three structural proteins (C, prM/M, E) and seven non-structural proteins (NS1, NS2A, NS2B, NS3, NS4A, NS4B and NS5) [10]. Within each serotype, genotypes can be phylogenetically classified on the basis of their E gene; DENV-1 includes six genotypes (I, II, III (sylvatic), IV, V, and VI); DENV 2 includes six genotypes (Asian I, Asian II, Asian/American, American, Cosmopolitan, and sylvatic); DENV-3 includes five genotypes (I, II, III, IV, and V); DENV-4 includes five genotypes (I, IIA, IIB, III, and sylvatic) [2, 11]. Previous studies have suggested that the transmission of the various serotypes is cyclic, with distinct serotypes periodically re-emerging to dominate, and the introduction of new serotypes or genotypes leading to new epidemics or outbreaks [11–15]. However, the circulating patterns of viruses during infection in human hosts and mosquito vectors are still unclear.

In Thailand, the first case of dengue disease was reported in 1949 and the first major outbreak of severe dengue (dengue hemorrhagic fever, DHF) was documented in Bangkok (central region) in 1958 [2, 11]. Dengue affects inhabitants throughout the country and has become a major public health problem annually [2, 16]. Although the dengue situation in northeastern Thailand has become critical in recent years, with both rising case numbers and increasing disease severity, there is no information available on the DENV genotype in this region.

In the present study, we performed molecular characterization of DENV detected in humans and *Aedes* mosquitoes in order to explore, for the first time, the epidemiological relationship between humans and mosquitoes and molecular characteristics of circulating DENV strains in northeastern Thailand.

## Materials and methods

### Study site, recruitment, and sample collection

**Fig 1:**
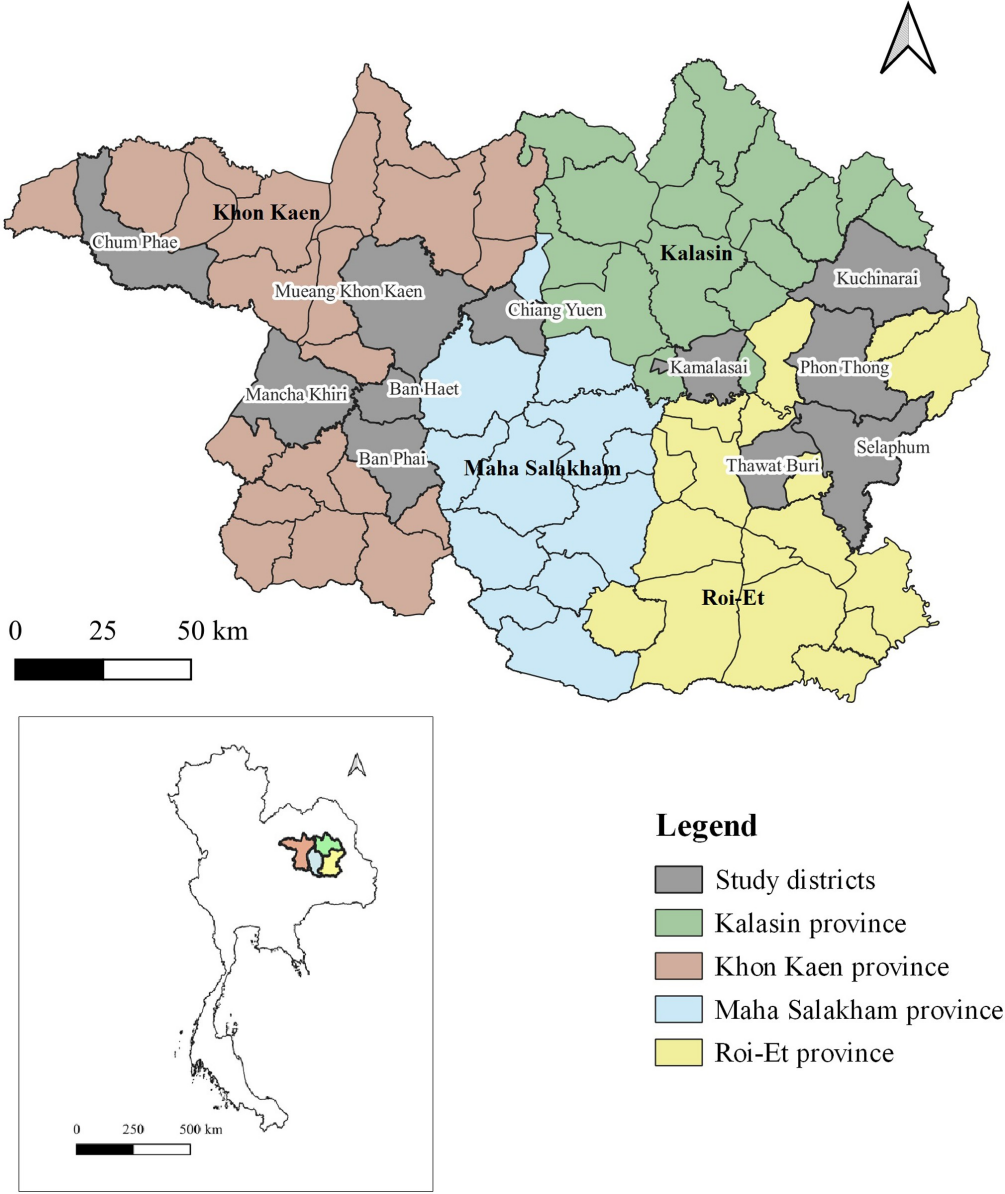
Map of the study area. Sample collections were done in four provinces of northeastern Thailand which are highlighted in green, brown, blue, and yellow. The locations of study districts are marked on the map in gray. The map was created using QGIS 3.16 software.

Plasma and mosquito samples obtained from a prospective, hospital-based, case-control study in northeastern Thailand [8] were used. The study was conducted in four provinces in northeastern Thailand from June 2016 to December 2018 (Fig 1). Eleven district hospitals in these provinces were included: Mancha Khiri (16° 12’ N 102° 29’ E), Chum Phae (16° 34’ N 102° 5’ E), Ban Phai (16° 3’ N 102° 43’ E), Ban Haet (16° 12’ N 102° 46’ E), and Mueang Khon Kaen (16° 25’ N 102° 49’ E) district hospitals in Khon Kaen Province; Selaphum (16° 2’ N 103° 59’ E), Phon Thong (16° 18’ N 103° 57’ E), and Thawat Buri (16° 1’ N 103° 45’ E) district hospitals in Roi Et Province; Kamalasai (16° 18’ N 103° 36’ E) and Kuchinarai (16° 30’ N 104° 1’ E) district hospitals in Kalasin Province; and Chiang Yuen (16° 25’ N 103° 3’ E) district hospital in Maha Sarakham Province.

The methodology of sample collection was previously described [8]. Briefly, plasma samples were collected from patients presenting with dengue-like symptoms, with potential dengue infections based on the presence of fever (> 38°C), no recent travel history during the previous 7 days, and being older than five years of age.

For the entomological investigation, adult *Aedes* mosquitoes were collected from the patient’s household and four additional neighboring houses within a 100-meter radius of the patient’s house using portable Prokopack aspirators [17]. The collection was conducted for 15 minutes indoors (mainly in bedrooms and living rooms) and 15 minutes outdoors near the house (primarily around human-made articles, backyard/patio, vegetation, etc). Mosquito sex and species (*Ae*. *aegypti* or *Ae*. *albopictus*) were identified under a stereomicroscope using morphological keys [18, 19]. *Aedes* mosquitoes from the patient’s house and neighboring houses were designated as being from one combined collection cluster.

### Confirmation of dengue virus infection in humans

During the study period, we obtained 292 plasma samples from suspected dengue patients (S1 Fig). The preliminary screening for DENV in the patient’s plasma was performed using the SD BIOLINE Dengue Duo kit (Standard Diagnostics, Suwon, Korea), according to the manufacturer’s instructions. Laboratory-confirmed DENV detection and serotyping were carried out in the laboratory at Khon Kaen University, Khon Kaen province, Thailand. Briefly, viral RNA was extracted from 140 μl of each plasma sample using QIAamp Viral RNA Mini Kits following the manufacturer’s instructions (Qiagen, Germany), and DENV detection and serotyping were performed using qRT-PCR as described by Shu et al. [20].

### DENV detection in adult mosquitoes

A total of 192 female *Aedes* mosquito pools containing 902 *Aedes* mosquitoes (1–32 mosquitoes per pool) were processed for DENV identification by qRT-PCR (S1 Fig). These 192 pools originated from different collection clusters. Mosquito abdomens were separated from the head-thorax and pooled. These pooled and individual head-thorax samples were triturated, using sterile pestles and 1.5 ml Eppendorf tubes (Eppendorf AG, Hamburg, Germany) in 500 μl of Leibovitz’s L-15 medium (Gibco, Thermo Fisher Scientific, USA). The resulting suspension was clarified by centrifugation 800×g at 4°C for 5 minutes. Next, 140 μl of each sample was transferred to Eppendorf tubes for RNA extraction, and the remaining suspensions were stored at −80°C. Viral RNA extraction was performed by using QIAamp Viral RNA Mini Kits following the manufacturer’s instructions (Qiagen, Germany). Mosquito samples were subjected to two rounds of PCR for DENV detection and serotyping using the primer set of Lanciotti et al. [21] with minor modifications to perform it on real-time PCR. The viral RNA was reverse-transcribed into cDNA by specific D2 primer synthesized using SuperScript III first-strand synthesis system (Invitrogen, USA) according to the manufacturer’s instructions and cDNA was stored at -20°C until used. The first round of PCR aimed to screen DENV infection from mosquito pooled samples using SYBR green-based real-time and the primer set of Lanciotti et al. [21]. All individual mosquito specimens from each PCR-positive pool were subjected to the second round of PCR for DENV serotyping by SYBR green-based real-time PCR using the primer set of Lanciotti et al. [21]. The PCR reaction comprised pre-denaturation at 95°C for 2 minutes followed by 35 cycles at 95°C for 15 seconds, 55°C for 30 seconds, and a final period of 72°C for 42 seconds. The PCR amplification used an Applied Biosystems® 7500 Real-Time PCR machine (Applied Biosystems, CA, USA). Positive control and negative control (without cDNA template) were included with each amplification reaction. The resulting data was analyzed using the software provided by Applied Biosystems based on the Tm and Ct amplification plot values. The positive results were re-confirmed by visualization on 2% agarose gel electrophoresis staining with ethidium bromide.

### Serotype matching between dengue patients and mosquitoes

To evaluate the relationship of DENV infection between patients and mosquitoes in the same residential area, DENV serotypes from 75 patients’ plasma were matched with those from mosquitoes collected from the collection clusters around each patient’s residence (S1 Fig). Where the patient’s plasma had at least one serotype in common with the mosquito pool, a match was assigned. On the other hand, if there was no DENV serotype in common between the patient’s plasma sample and the mosquito pool, or if the latter was negative for DENV, this was assigned as a serotype mismatch.

### DENV envelope gene sequencing

Full-length E gene PCR products were directly amplified from patient plasma and mosquito pooled samples to avoid genetic changes due to the culture process. The viral RNA was extracted from the samples using QIAamp Viral RNA Mini kit (Qiagen, Germany) according to the manufacturer’s instructions. cDNA fragments covering the E gene of DENV were synthesized by specific primers of each serotype as previously described [22] using SuperScript III first-strand synthesis system (Invitrogen, USA) according to the manufacturer’s instructions and stored at -20°C until use. Two overlapping fragments (F and L fragments) of the E gene were amplified primers as previously described [22], using PrimeStar GXL DNA polymerase (Takara, Japan) according to the manufacturer’s instructions. The PCR amplification cycles were performed with a step of initial DNA denaturation at 98°C for 5 minutes followed by 35 cycles of denaturation at 98°C for 10 seconds and annealing at 55°C for 15 seconds and extension at 68°C for 90 seconds. The specific amplicons were purified from 0.8% agarose gel by gel cutting and extraction using the QIAquick gel extraction kit (Qiagen, Germany). Purified amplicons of each sample were subjected to Sanger sequencing by using cycle sequencing reactions and dye terminator methodologies of Macrogen company (Macrogen, Seoul, Korea) using four overlapping primers for each serotype as previously described [22].

### Phylogenetic analysis

The phylogenetic analysis based on the nucleotide sequence of the envelope gene-encoding region of the DENV was constructed and used to elucidate the origins of disease outbreaks. The E gene sequences obtained in this study were aligned with other DENV sequences from those previously isolated in Thailand and neighboring countries and from other regions of the world available in the GenBank database (www.ncbi.nlm.nih.gov) using ClustalW in Bioedit. Maximum-likelihood trees were constructed in MEGA X using 35 reference sequences for DENV-1, 65 sequences for DENV-2, and 40 sequences for DENV-4 (S2 Table) with the best-fit nucleotide substitution model (TN93+G). Bootstrap values were done with 1000 replications. Genotypic classification of DENV-1, -2, and -4 followed Goncalvez et al. [23], Twiddy et al. [24] and AbuBakar et al. [25] respectively.

### Accession numbers

The full-length E gene sequences obtained in this study were deposited in GenBank and granted accession numbers MT524489 to MT524513 (Table 1).

**Table 1:** Information on dengue virus strains in this study.

Sample code	Host	Location (District, Province)	Collection date	Serotype	Genotype	Accession No.
D1H/4024-01/16	Human	Ban Haet, Khon Kaen	Jun-2016	1	I	MT524490
D1H/4024-02/16	Human	Ban Haet, Khon Kaen	Jun-2016	1	I	MT524493
D1H/4024-07/16	Human	Ban Haet, Khon Kaen	Jun-2016	1	I	MT524492
D1M/4024-07/16	Mosquito	Ban Haet, Khon Kaen	Jun-2016	1	I	MT524491
D1M/4024-08/16	Mosquito	Ban Haet, Khon Kaen	Jun-2016	1	I	MT524494
D1H/4010-02/16	Human	Ban Phai, Khon Kaen	Sep-2016	1	I	MT524489
D1H/4405-08/17	Human	Chiang Yuen, Maha Salakham	Jul-2017	1	I	MT524496
D1H/4603-24/18	Human	Kamalasai, Kalasin	Aug-2018	1	I	MT524495
D2H/4005-02/16	Human	Chum Phae, Khon Kaen	Jun-2016	2	Asian I	MT524502
D2H/9918-02/18	Human	Mueang khon kaen, Khon Kaen	May-2018	2	Cosmopolitan	MT524498
D2H/4603-10/18	Human	Kamalasai, Kalasin	Jun-2018	2	Cosmopolitan	MT524499
D2H/4603-11/18	Human	Kamalasai, Kalasin	Jun-2018	2	Asian I	MT524501
D2H/4405-26/18	Human	Chiang Yuen, Khon Kaen	Jun-2018	2	Cosmopolitan	MT524500
D2H/9918-09/18	Human	Mueang khon kaen, Khon Kaen	Aug-2018	2	Cosmopolitan	MT524497
D4H/4005-06/16	Human	Chum Phae, Khon Kaen	Jun-2016	4	I	MT524505
D4M/4005-07/16	Mosquito	Chum Phae, Khon Kaen	Jun-2016	4	I	MT524506
D4H/4507-09/16	Human	Phon Thong, Roi Et	Jul-2016	4	I	MT524504
D4M/4510-08/16	Mosquito	Selaphum, Roi-Et	Aug-2016	4	I	MT524503
D4H/4005-11/16	Human	Chum Phae, Khon Kaen	Aug-2016	4	I	MT524508
D4H/4005-15/16	Human	Chum Phae, Khon Kaen	Aug-2016	4	I	MT524507
D4H/4507-15/16	Human	Phon Thong, Roi Et	Aug-2016	4	I	MT524510
D4H/4507-18/16	Human	Phon Thong, Roi-Et	Sep-2016	4	I	MT524509
D4H/4507-19/16	Human	Phon Thong, Roi-Et	Sep-2016	4	I	MT524512
D4H/4005-29/16	Human	Chum Phae, Khon Kaen	Oct-2016	4	I	MT524513
D4H/4605-01/17	Human	Kuchinarai, Kalasin	Jan-2017	4	I	MT524511

Samples were named in a format consisting of “DENV serotype host/sample ID/Year of sample collection”.

### Ethics statement

The human samples in the present study were obtained from a dengue case-control study [8]. The present study was approved by the Khon Kaen University Ethics Committee for Human Research (KKUEC, no. HE611454). Informed consent was obtained in writing from all participants before sample collection.

## Results

### DENV serotype distribution in human samples

A total of 103 of 292 plasma samples were positive for DENV (S1 Fig). Serotype identification revealed all four DENV serotypes circulated in these samples. Of the 103 DENV positive samples, single DENV serotypes indicating mono-infection were detected in 79 (77%) samples, while multiple serotypes indicating co-infection were found in 24 (23%) samples. The predominant DENV serotypes in 2016 and 2018 were DENV-4 (74%) and DENV-3 (53%), respectively, whereas in 2017 DENV-1, -3, and DENV-4 were found with equal prevalence (Table 2).

**Table 2:** Dengue virus infection and serotype distribution in human blood and individual mosquitoes per year.

2016						
Sample	No. of samples	DENV positive	DENV-1	DENV-2	DENV-3	DENV-4
Human blood	133	42	9 (21%)	3 (7%)	9 (21%)	31 (74%)
*Ae*. *aegypti*	337	77	8	18	56	7
*Ae*. *albopictus*	9	4	0	1	3	1
Total *Aedes*	346	81	8 (10%)	19 (23%)	59 (73%)	8 (10%)
**2017**						
Sample	No. of samples	DENV positive	DENV-1	DENV-2	DENV-3	DENV-4
Human blood	35	8	3 (38%)	0	3 (38%)	3 (38%)
*Ae*. *aegypti*	137	4	1	3	3	0
*Ae*. *albopictus*	0	0	0	0	0	0
Total *Aedes*	137	4	1 (13%)	3 (38%)	3 (38%)	0
**2018**						
Sample	No. of samples	DENV positive	DENV-1	DENV-2	DENV-3	DENV-4
Human blood	124	53	15 (28%)	22 (42%)	28 (53%)	4 (8%)
*Ae*. *aegypti*	417	64	9	50	37	2
*Ae*. *albopictus*	2	0	0	0	0	0
Total *Aedes*	419	64	9 (17%)	50 (94%)	37 (70%)	2 (4%)

Note: The number of DENV serotypes distribution reported in the table consists of mono-infected and co-infected cases.

### DENV serotype distribution in mosquito samples

A total of 902 female *Aedes* individual mosquitoes (contained in 192 pools) were captured during the study period (Table 2). The predominant mosquito species was *Ae*. *aegypti* (98.8%) followed by *Ae*. *albopictus* (1.2%) (Table 2). One hundred forty-nine (16.5%) of the 902 female mosquitoes were positive for DENV. Serotyping of individual mosquito head-thoraces revealed that the predominant DENV serotypes were DENV-3 (73%) in 2016, DENV-2 and DENV-3 (38% of each serotype) in 2017, and DENV-2 (94%) in 2018 (Table 2). Of the 149 DENV positive individual mosquito samples, single DENV serotypes indicating mono-infection were detected in 105 (70%) samples, while multiple serotypes indicating co-infection were found in 44 (30%) samples.

### Relationships of DENV infection between dengue patients and mosquitoes from the same residence area

**Fig 2:**
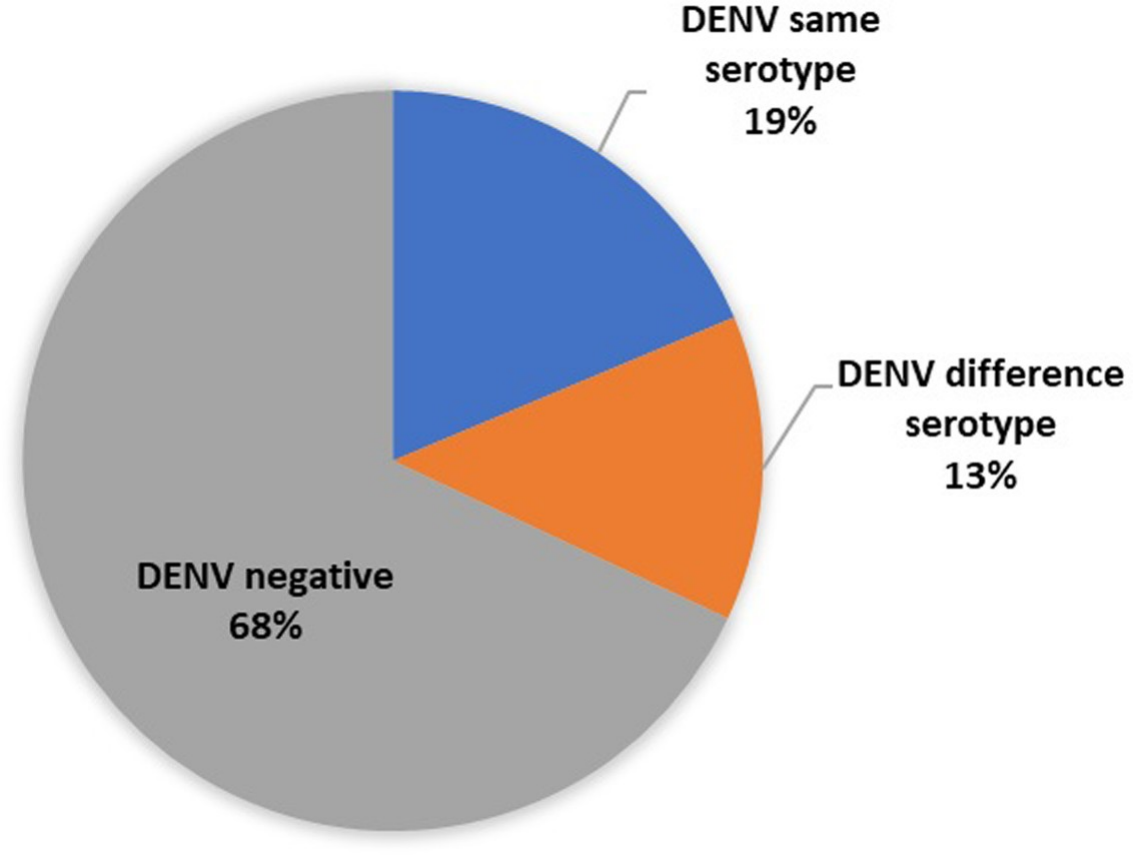
Serotypes in 75 DENV-positive patients from households where corresponding DENV-positive mosquitoes were collected. In 14 households (19%), the serotype in patients and mosquitoes matched each other, i.e. were the same (blue); in 10 households (13%), the serotypes in patients and mosquitoes were the different (orange); and in 51 households (68%) patients were DENV-positive whereas mosquitoes were DENV-negative (grey). More details of the molecular characteristics of DENV-positive patients and mosquitoes are provided in S3 Table.

Out of 103 DENV-positive patients, mosquito collection was done only in 75 patient’s residential areas. DENV serotypes in plasma of those patients were compared with DENV serotypes from the infected mosquitoes from their residences. Of the 75 plasma samples, 14 (19%) matched a serotype from the DENV positive mosquitoes samples (Fig 2). Among 61 (81%) mismatched samples, 10 (13%) had a different DENV serotype in the corresponding mosquito samples, while 51 (68%) corresponded to DENV negative mosquito samples. The molecular characteristics of 24 pairs out of 75 paired samples are presented in S3 Table.

### Phylogenetic analysis

Sequencing of the E gene was successful for 25 samples, 21 from patients, and 4 from mosquito pools. They contained eight DENV-1, six DENV-2, and 11 DENV-4 sequences (Table 1).

**Fig 3:**
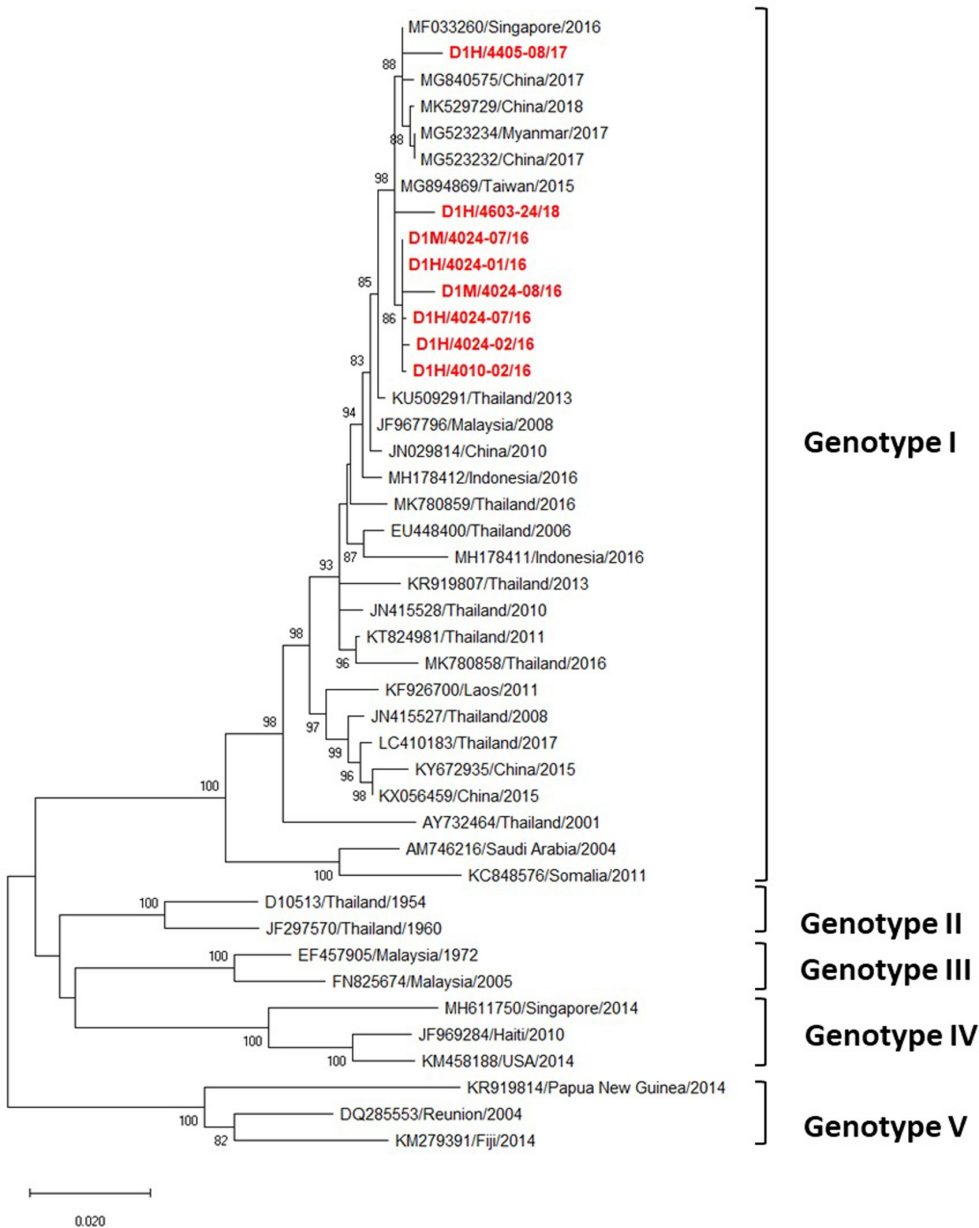
Phylogenetic analysis of DENV-1. Maximum-likelihood tree of the E gene sequences of DENV-1 was generated in the MEGA X program using the TN93 + G model with 1000 bootstrap replications. Bootstrap support values exceeding 80% are shown on branch nodes. The virus names of each sequence retrieved from the NCBI database are labeled as follows: GenBank accession number/country/isolated year. Samples collected in the present study are in red bold font. Annotation on the right denotes DENV genotype.

Phylogenetic analysis of DENV-1 following Goncavez’s classification [23] showed that all eight DENV-1 strains were genotype I (Fig 3), and that all eight were from the same clade. Seven strains were closely related to the DENV-1 genotype I isolated from Taiwan in 2015, the other (D1H/4405-08/17) being closely related to DENV-1 isolated between 2016 and 2018 from other Asian countries including Singapore in 2016, China in 2017–18 and Myanmar in 2017 (Fig 3).

**Fig 4:**
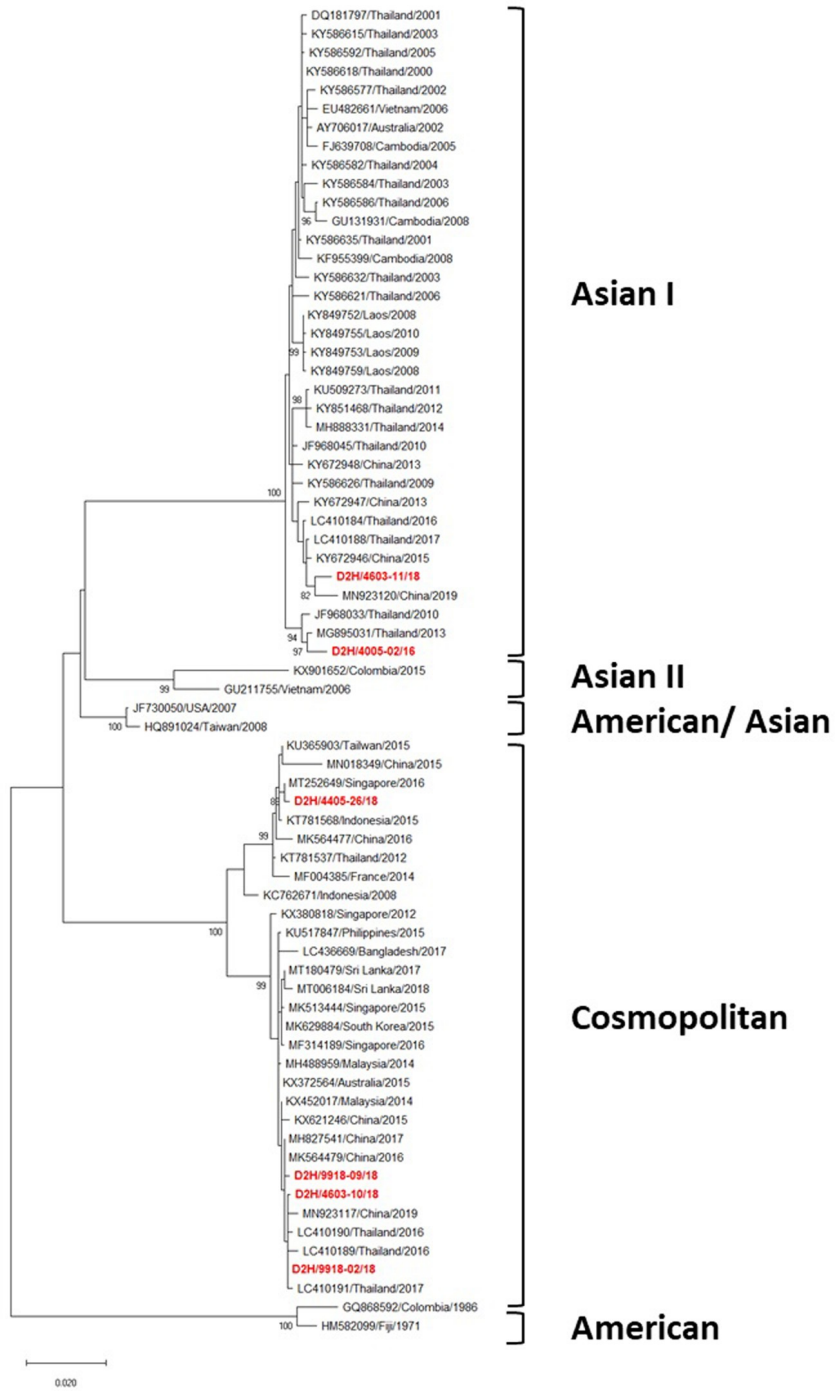
Phylogenetic analysis of DENV-2. Maximum-likelihood tree of the E gene sequences of DENV-2 was generated in the MEGA X program using the TN93 + G model with 1000 bootstrap replications. Bootstrap support values exceeding 80% are shown on branch nodes. The virus names of each sequence retrieved from the NCBI database are labeled as follows: GenBank accession number/country/isolated year. Samples collected in the present study are in bold red font. Annotation on the right denotes DENV genotype.

Of the six DENV-2 strains, two were classified as Asian I and four as Cosmopolitan genotypes [24] (Fig 4). In turn, the Asian I genotype was clustered into two clades. The Asian I strain D2H/4005-02/16 was closely related to the DENV-2 Thai strain isolated in 2013, while the other (D2H/4603-11/18) was closely related to one isolated in Thailand in 2017, and in China in 2015 (Fig 4). The four Cosmopolitan strains also clustered into two distinct clades. Three Cosmopolitan strains (D2H/4603-10/18, D2H/9918-02/18, and D2H/9918-09/18) were closely related to ones isolated in Thailand and China during 2016–2017, whereas the remaining strain (D2H/4405-26/18) was closely related to DENV-2 isolated from Singapore in 2016 (Fig 4).

**Fig 5:**
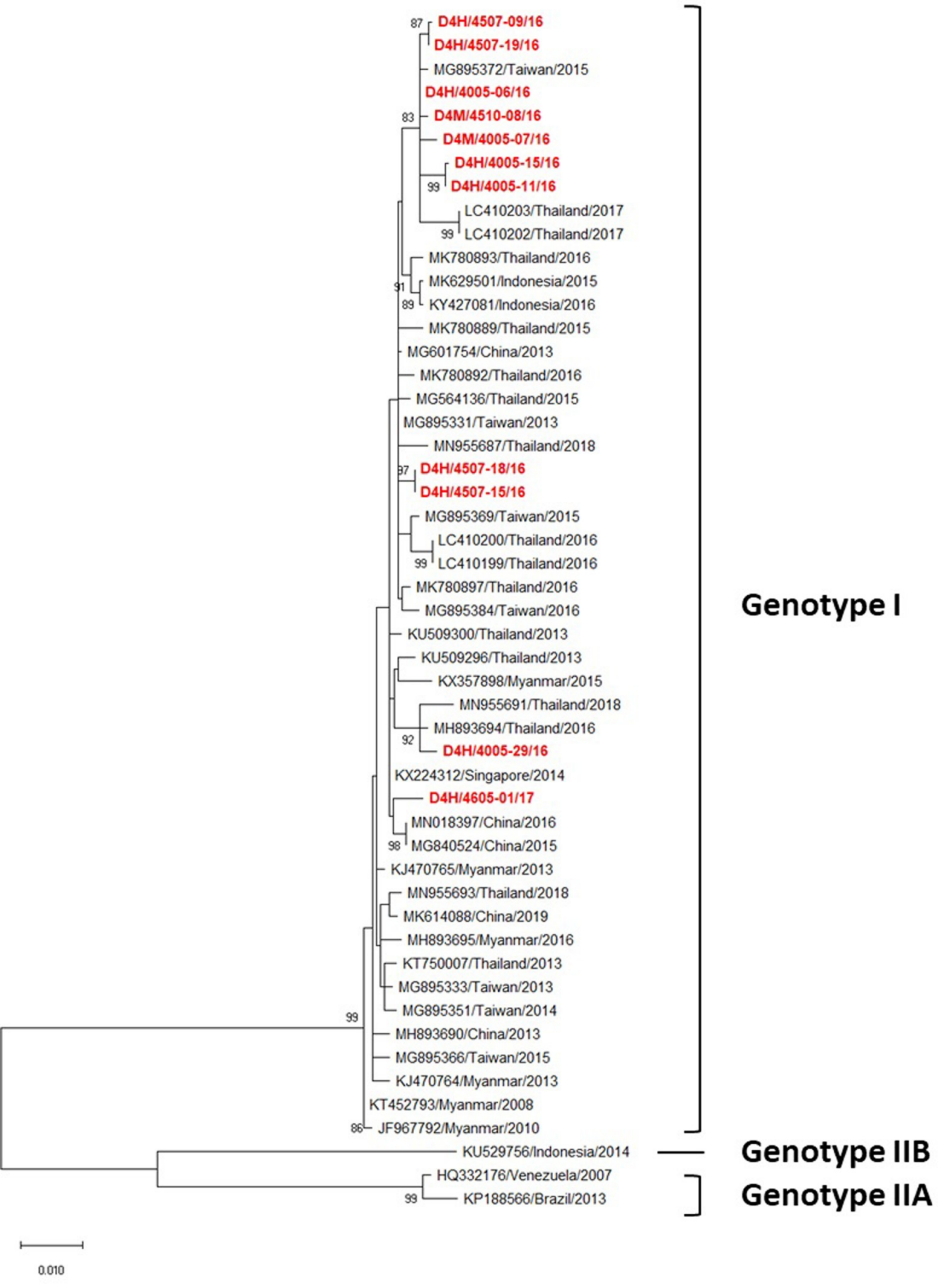
Phylogenetic analysis of DENV-4. Maximum-likelihood tree of the E gene sequences of DENV-4 was generated in the MEGA X program using the TN93 + G model with 1000 bootstrap replications. Bootstrap support values exceeding 80% are shown on branch nodes. The virus names of each sequence retrieved from the NCBI database are labeled as follows: GenBank accession number/country/isolated year. Samples collected in the present study are in red bold font. Annotation on the right denotes DENV genotype.

Phylogenetic analysis of DENV-4 following AbuBakar et al. [25] revealed that all 11 DENV 4 strains obtained in this study belonged to genotype I (Fig 5). However, they were clustered into four distinct clades (Fig 5). Seven strains from 2016 were closely related to one isolated in Taiwan in 2015 and in Thailand in 2017, whereas two strains collected during 2016 (D4H/4507-15/16 and D4H/4507-18/16) were closely related to one isolated in Taiwan in 2013. The two remaining strains were closely related to ones found in Thailand and neighboring countries, e.g. Singapore in 2014 and China in 2015 to 2016 (Fig 5).

## Discussion

Recent evidence indicates that the dengue outbreak situation in northeastern Thailand has become critical, causing annually peaks of the disease associated with increasing morbidity and mortality humans [2]. So far, there is limited information on the molecular characteristics of DENV in this region. The present study is the first to describe the relationship of DENV infection between humans and mosquitoes, and molecular characteristics of DENV in northeastern Thailand.

Approximately one third (35.3%) of dengue-suspected patients recruited in this study had a confirmed DENV infection, indicating a high burden of dengue in 2016 and 2018 (Table 2). All four DENV serotypes were found co-circulating during the three years of the study. These findings are similar to those from other regions of Thailand during the same period [11, 16]. The most prevalent serotypes in 2016 and 2018 were DENV-4 and DENV-3 respectively, whereas, DENV-1, -3, -4 were equally prevalent in 2017. With the lack of molecular data on DENV in northeastern Thailand, we were not able to compare this serotype profile to those of previous years. During the three years (June 2016 to December 2018) of our study, Thailand faced dengue outbreaks in 2016 and 2018 in several provinces [26, 27]. Fewer dengue cases in 2017 were also reported by national surveillance (The Office of Disease Prevention and Control Region, ODPC). In addition, a previous report also showed that, prior to our study, during 2013–2015, the yearly distribution of DENV serotypes in Thailand included a high prevalence of DENV-2 [27]. This could have resulted in fewer DENV-2 human cases in 2017 due to recently acquired prior immunity. However, DENV-2 is still present in the collected mosquitoes at the same time, it is common to find all serotypes circulating in vectors or hosts, given the hyperendemicity of DENV in Thailand. The virus can also be directly transmitted to the next vector generation by vertical transmission. Similarly, a study conducted in India observed that the DENV-2 disappeared in 2016 after predominating between 2011–2015 [28].

Dengue virus detection in mosquitoes during an outbreak has been suggested as a potential tool for early warnin and designing effective vector control strategies [29, 30]. We collected *Aedes* mosquitoes from houses of suspected dengue patients in northeastern Thailand, the most prevalent species was *Ae*. *aegypti* (98.8%) followed by *Ae*.*albopictus* (1.2%) (Table 2). Our study found 16.5% (149/902) of mosquitoes were DENV-positive. Similarly, in southern Thailand, 16% of field-caught *Aedes aegypti* and 36.2% of *Aedes albopictus* were infected with DENV during the early rainy season of 2005 [31]. Several studies have described DENV infection in field-caught *Aedes* mosquitoes, with various infection rate including 10.8% in Brazil [32], 12.7% and 62% in Colombia [33, 34], 2.8% in Philippines [35]. These findings suggested that the infection rate of virus in field-caught mosquitoes is highly variable, possibly depending on the design and conditions of each study [36, 37]. Serotyping results illustrated that all four serotypes were circulating in the mosquitoes in this region. DENV-3 and DENV-2 were predominant in 2016 and 2018 respectively, while both serotypes were most prevalent in 2017.

Moreover, we found co-infection of different DENV serotypes: 23% and 30% in human and mosquito samples respectively. This finding is consistent with several studies which reported the concurrence of DENV infection in the same patient with multiple serotypes at different percentages ranging from 3% to 43% [38–41]. Similar observations of co-infection with multiple DENV serotypes have been reported in other countries and also in Thailand [31, 42, 43]. All four serotypes circulating in the region have been found in combinations within single patients [41]. Several factors might be involved in co-infection including: i) the multiple feeding behavior of *Ae*. *aegypti*, which feeds more than once during its genotropic cycle [31, 41, 42], ii) the high attack rates of cases during dengue epidemics which may result in many infections with multiple serotypes in humans, and also provide opportunities for mosquitoes to become infected with multiple serotypes [42], iii) transovarial transmission of dengue viruses in mosquitoes [31], iv) asymptomatic cases as a source of infection in mosquitoes [42].

Results from the present study, show the difference of serotype distribution in hosts and vectors by year, and suggest that mosquitoes may serve as a maintenance mode for the virus in the environment. When the immunity level in human population to a certain serotype decreases, a burst of infections may follow [32]. Moreover, our results are consistent with previous studies which showed specific DENV serotypes building up in mosquitoes, then becoming predominant in humans in the subsequent year [32, 33].

Although several studies have evaluated the presence of DENV-infected *Aedes* mosquitoes in all four regions of Thailand including central [44–49], southern [31], northern [47, 50], and northeastern [51], our study is the first to target the residential area of DENV-infected patients within the onset of symptoms in northeastern Thailand. The DENV serotypes of patient samples were compared with mosquito samples collected from the patient’s residential area within a 100-meter radius. About one fifth (19%) of the patients had a serotype matching the serotype in the mosquitoes collected, while remaining 81% were serotype mismatched, consistsing of 13% with a different serotype and another 68% whose corresponding mosquitoes were DENV negative (Fig 2). This result suggests that transmission outside the residence may be important. Since most of the cases were children and adults (S4 Table) who have spent day time outside their residence (e.g., school, worksite/office), it is possible that they were exposed there to infectious mosquitoes. This finding is consistent with previous studies conducted in Brazil and Philippines that found differences in DENV serotypes between patients and mosquitoes in the same home [32, 35]. Previous reports suggest that DENV infection often occurs elsewhere, other than the home, e.g. workplace, school or college, temple or farm field [9, 35, 52]. Numerous factors may influence these results including: i) asymptomatic infections with different DENV serotypes in members of the same household, ii) transovarial transmission of DENV in the household involving serotypes to which the residents have recent prior immunity [35]. It is essential to note that spending time in an area or work environment during the day that is infested with *Aedes* mosquitoes may increase the risk of dengue infection. This supports the idea that DENV transmission is probably driven by the movement of infected humans [35].

The phylogenetic analysis revealed that all eight DENV-1 strains were classified as genotype I (Fig 3), which mainly circulates in Asian countries especially Thailand [11, 16, 53–56]. Interestingly, these DENV-1 strains were not grouped within the same clade as Thai strains isolated during the same period in northern [11] and southern [16] regions. Rather, those other strains were closely related to strains from other Asian countries during 2015–2018, such as a Taiwanese strain isolated from a traveler who returned from Thailand in 2015, a Singaporean strain in 2016, a Myanmar strain in 2017, and Chinese strains in 2017–2018. This indicates that DENV-1 circulating strains in northeastern Thailand are likely to have been imported from other Asian countries (Fig 3).

We observed DENV-2 Asian I and Cosmopolitan genotypes co-circulating in this region (Fig 4), as they commonly do elsewhere in Asia, especially Southeast Asia (SEA) [11, 14, 16, 57–59]. The Asian I strains were clustered into different 2 clades. Interestingly, the strain D2H/4005-02/16 was closely related to the Taiwanese strain isolated from two travelers returning from Thailand in 2010 [60] and 2013 [61], suggesting they were likely to have circulated for at least 3–4 years in northeastern Thailand. Moreover, the remaining strain D2H/4603-11/18 was closely related to one isolated in Guangzhou (China) during 2019, suggesting they have a common ancestor. Therefore, northeastern Thailand seems to be a potential source of dengue transmission to other parts of Asian countries (Fig 4). The four Cosmopolitan strains were clustered into two clades. Three strains (D2H/4603-10/18, D2H/9918-02/18, and D2H/9918-09/18) were closely related to ones isolated during the same period in the central and northern regions of the country, and in other Asian countries such as China and Singapore (Fig 4). This demonstrates that the DENV-2 northeastern strains were not only transferred from other parts of Thailand in the same period but also were persistent in this region (Fig 4).

All 11 DENV-4 strains were clustered into genotype I, which commonly circulates and was recently found in Thailand [11, 16, 62] (Fig 5). Although all strains were grouped into this single genotype, they were clustered into 3 different clades (Fig 5). Nine strains were closely related to ones isolated in Thailand, Taiwan, and China during 2015–2016, with the remaining two (D4H/4507-15 and D4H/4507-18) being closely related to one isolated in Taiwan in 2013 from a traveler who returned from Thailand (Fig 5). This demonstrates that DENV-4 strains circulating in this region were not only transferred from other regions of Thailand in the same period but also imported from Southeast Asian countries (Fig 5). Interestingly, all seven DENV-4 northeastern strains collected during 2016 were closely related to the LC410202-03/Thailand/2017 strain which was isolated from the central region of Thailand in 2017 [11]. These results suggest that the northeastern region is a locus of active circulation of DENV genotypes, with the potential to export them not only to other regions of the country but also other countries around the world especially SEA through the movement of infected persons and mosquitoes [10].

Our study has some limitations, in particular in terms of generalizability (extrapolation). First, we defined inconsistent serotyping results as a mismatch, which included both the occurrence of different genotypes, and an infection linked to negative sample. Since negative was assigned as mismatched, it could increase the chance of mismatch due to the low titer of DENV in mosquitoes. Second, our mosquito collection focused on households, but transmission could occur in other locations where patients spend their daytime such as schools, work offices, and community centers [8]. Last, we were unable to sequence any DENV-3 viruses. Since our study performed E gene sequencing directly from the sample without virus isolation, to avoid working on passaged viruses, this gap might be attributed to low viral titers which were insufficient for conventional PCR amplification. Shorter fragments of PCR products have been suggested to be more suitable for sequencing [10], and this is being considered for our future studies.

In conclusion, our study provides the first data on molecular characteristics of DENV in northeastern Thailand during 2016 to 2018. Our data confirmed the hyperendemicity of all four DENV serotypes in northeastern Thailand. The low proportion of matches between DENV serotypes in patients and mosquitoes from the patients’ residence areas suggests that non-residential transmission may be important. Our phylogenetic analysis revealed that the virus circulating in this region shared high homology with the virus from other regions of Thailand and Southeast Asian countries in the same period, as well as with persisting strains that originated in Thailand and other southeast Asian countries. These findings indicated that human movement has an important role in infection and disease transmission in the region. Taken together, our findings highlight that vector control, and early warning based on DENV detection in vectors, are important strategies for the prevention of dengue epidemics in northeastern Thailand. Continuous molecular surveillance of DENV in northeastern Thailand to observe the virus replacement and also better understand dengue transmission dynamics in this region is required.

## Supporting information

S1 Fig The diagram of the number of the samples used in this study. (TIF)

S1 Table Primer used for envelope (E) gene fragment amplification and sequencing in the present study. (DOCX)

S2 Table Published sequences used in this study for phylogenetic analysis. (accessed from GenBank, the National Centre for Biotechnology Information https://www.ncbi.nlm.nih.gov/genbank/).
(DOCX)

S3 Table Molecular characteristic of dengue virus between patient and adult mosquitoes collected from patient’s resident area. The molecular characteristics of 24 pairs of DENV positive samples were shown in this table. Gray row label represents serotype matched between patients and mosquitoes from their resident area.
(DOCX)

S4 Table Age of 75 dengue patients who have mosquitoes inside the resident area. (DOCX)
