# Supplementary figures and images for: Dengue virus in humans and mosquitoes and their molecular characteristics in northeastern Thailand 2016-2018
Source: PLoS One. 2021 Sep 14;16(9):e0257460. doi: 10.1371/journal.pone.0257460 (PMC8439490; doi:10.1371/journal.pone.0257460)

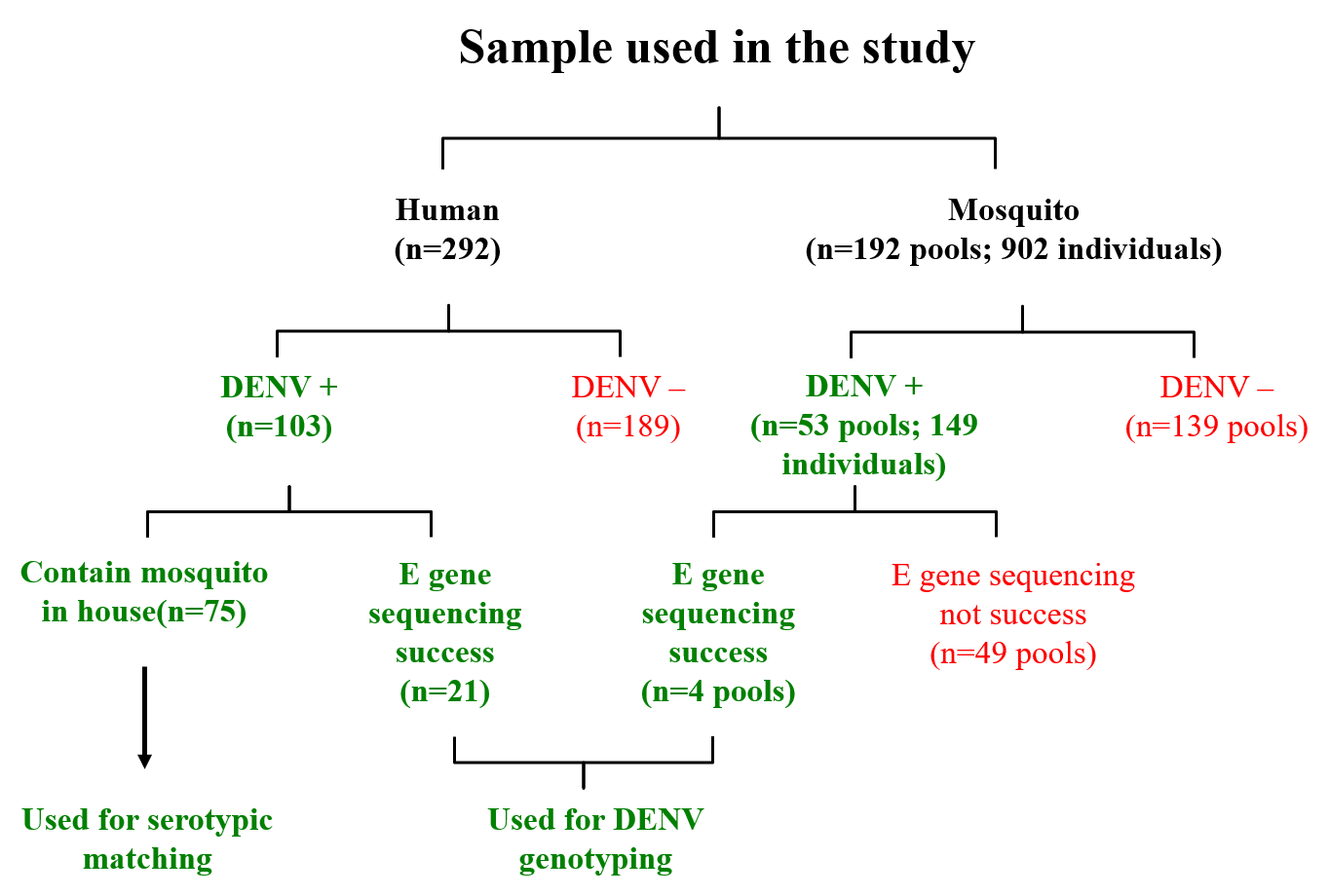

Supplement: S1 Fig — (TIF) [file pone.0257460.s001.tif]
