# Supplementary material for: Dengue virus in humans and mosquitoes and their molecular characteristics in northeastern Thailand 2016-2018
Source: PLoS One. 2021 Sep 14;16(9):e0257460. doi: 10.1371/journal.pone.0257460 (PMC8439490; doi:10.1371/journal.pone.0257460)
Supplement: S1 Table — (DOCX) [file pone.0257460.s002.docx]

**S1 Table. Primer used for envelope (E) gene fragment amplification and sequencing in the present study.**

| **Primer** | **Fw primer sequence (5’-3’)** | **Rv primer sequence (5’-3’)** | **Amplicon size (bp)** | **Reference** |
| --- | --- | --- | --- | --- |
| DENV-1 | GAGACACCCAGGATTCACGG (820-839) | TCCAAGGCAGTGGTAGGTCT (1595-1576) | 777 | The present study |
|  | GCAACCATAACACCTCAA (1418-1435) | TGGCTGATCGAATTCCACAC (2600-2581) | 1183 | Warrilow et al., 2012 |
| DENV-2 | GAAACATGCCCAGAGAATTGAAACT (789 - 813) | CCCTTCATATTGTACTCTGATAACTATTGTTCC (1920-1888) | 1132 | Warrilow et al., 2012 |
|  | AAGCTTGGCTGGTGCACAGGCAATGGTT (1547- 1574) | GGGGATTCTGGTTGGAACTTGTATTGTTCTGTCC (2537-2504) | 991 |  |
| DENV-4 | TGTCATCGGAAGGAGCTTGG (746-765) | CTGGGATCCTAGCACTGTCAC (1680-1660) | 915 | The present study |
|  | CAATGGTTTTTGGACCTACCTCTACCATGG (1569-1598) | GGGGACTCTGGTTGAAATTTGTACTGTTCTGTCCA (2539-2505) | 971 | Warrilow et al., 2012 |

*DENV, dengue virus; Fw primer, Forward primer; Rv primer, Reverse primer. Numbering is based on DENV-1 strain DENV-1BR/90 (AF226685), DENV-2 strain New Guinea C (AF038403), DENV-4 strain Dominica 1981 (AF326573).
