# Supplementary material for: Dengue virus in humans and mosquitoes and their molecular characteristics in northeastern Thailand 2016-2018
Source: PLoS One. 2021 Sep 14;16(9):e0257460. doi: 10.1371/journal.pone.0257460 (PMC8439490; doi:10.1371/journal.pone.0257460)
Supplement: S2 Table — (accessed from GenBank, the National Centre for Biotechnology Information https://www.ncbi.nlm.nih.gov/genbank/). (DOCX) [file pone.0257460.s003.docx]

**S2 Table. Published sequences used in this study for phylogenetic analysis.**

(accessed from GenBank, the National Centre for Biotechnology Information <https://www.ncbi.nlm.nih.gov/genbank/>)

| **DENV serotype** | **Genotype** | **Accession number** | **Host**  **(isolation source)** | **Country** | **Year** |
| --- | --- | --- | --- | --- | --- |
| DENV-1 | I | MF033260 | *Homo sapiens* | Singapore | 2016 |
| DENV-1 | I | MG840575 | *Homo sapiens* | China | 2017 |
| DENV-1 | I | MK529729 | *Homo sapiens* | China | 2018 |
| DENV-1 | I | MG523234 | *Homo sapiens* | Myanmar | 2017 |
| DENV-1 | I | MG523232 | *Homo sapiens* | China | 2017 |
| DENV-1 | I | MG894869 | *Homo sapiens* | Taiwan | 2015 |
| DENV-1 | I | KU509291 | *Homo sapiens* | Thailand | 2013 |
| DENV-1 | I | KF967796 | *Homo sapiens* | Malaysia | 2008 |
| DENV-1 | I | JN029814 | *Homo sapiens* | China | 2010 |
| DENV-1 | I | MH178412 | *Homo sapiens* | Indonesia | 2016 |
| DENV-1 | I | MK788859 | *Homo sapiens* | Thailand | 2016 |
| DENV-1 | I | EU448400 | *Homo sapiens* | Thailand | 2006 |
| DENV-1 | I | MH178411 | *Homo sapiens* | Indonesia | 2016 |
| DENV-1 | I | KR919807 | *Homo sapiens* | Thailand | 2013 |
| DENV-1 | I | JN415528 | *Homo sapiens* | Thailand | 2010 |
| DENV-1 | I | KT824981 | *Homo sapiens* | Thailand | 2011 |
| DENV-1 | I | MK780858 | *Homo sapiens* | Thailand | 2016 |
| DENV-1 | I | KF926700 | *Homo sapiens* | Laos | 2011 |
| DENV-1 | I | JN415527 | *Homo sapiens* | Thailand | 2008 |
| DENV-1 | I | LC410183 | *Homo sapiens* | Thailand | 2017 |
| DENV-1 | I | KY672935 | *Homo sapiens* | China | 2015 |
| DENV-1 | I | KX056459 | *Homo sapiens* | China | 2015 |
| DENV-1 | I | AY732464 | *Homo sapiens* | Thailand | 2001 |
| DENV-1 | I | AM746216 | *Homo sapiens* | Saudi Arabia | 2004 |
| DENV-1 | I | KC848576 | *Homo sapiens* | Somalia | 2011 |
| DENV-1 | II | D10513 | *Homo sapiens* | Thailand | 1954 |
| DENV-1 | II | JF297570 | *Homo sapiens* | Thailand | 1960 |
| DENV-1 | III | EF457905 | *Homo sapiens* | Malaysia | 1972 |
| DENV-1 | III | FN825674 | *Homo sapiens* | Malaysia | 2005 |
| DENV-1 | IV | MH611750 | *Homo sapiens* | Singapore | 2014 |
| DENV-1 | IV | JF969284 | *Homo sapiens* | Haiti | 2010 |
| DENV-1 | IV | KM458188 | *Homo sapiens* | USA | 2014 |
| DENV-1 | V | KR919814 | *Homo sapiens* | Papua New Guinea | 2014 |
| DENV-1 | V | DQ285553 | *Homo sapiens* | Reunion | 2004 |
| DENV-1 | V | KM279391 | *Homo sapiens* | Fiji | 2014 |
| DENV-2 | Asian I | DQ181797 | *Homo sapiens* | Thailand | 2001 |
| DENV-2 | Asian I | KY586615 | *Homo sapiens* | Thailand | 2003 |
| DENV-2 | Asian I | KY586592 | *Homo sapiens* | Thailand | 2005 |
| DENV-2 | Asian I | KY586618 | *Homo sapiens* | Thailand | 2000 |
| DENV-2 | Asian I | KY586577 | *Homo sapiens* | Thailand | 2002 |
| DENV-2 | Asian I | EU482661 | *Homo sapiens* | Vietnam | 2006 |
| DENV-2 | Asian I | AY706017 | *Homo sapiens* | Australia | 2002 |
| DENV-2 | Asian I | FJ639708 | *Homo sapiens* | Cambodia | 2008 |
| DENV-2 | Asian I | KY586635 | *Homo sapiens* | Thailand | 2001 |
| DENV-2 | Asian I | KF955399 | *Homo sapiens* | Cambodia | 2008 |
| DENV-2 | Asian I | KY586632 | *Homo sapiens* | Thailand | 2003 |
| DENV-2 | Asian I | KY586621 | *Homo sapiens* | Thailand | 2006 |
| DENV-2 | Asian I | KY849752 | *Homo sapiens* | Laos | 2008 |
| DENV-2 | Asian I | KY849755 | *Homo sapiens* | Laos | 2010 |
| DENV-2 | Asian I | KY849753 | *Homo sapiens* | Laos | 2009 |
| DENV-2 | Asian I | KY849759 | *Homo sapiens* | Laos | 2008 |
| DENV-2 | Asian I | KU509273 | *Homo sapiens* | Thailand | 2011 |
| DENV-2 | Asian I | KY851468 | *Homo sapiens* | Thailand | 2012 |
| DENV-2 | Asian I | MH888331 | *Homo sapiens* | Thailand | 2014 |
| DENV-2 | Asian I | JF968045 | *Homo sapiens* | Thailand | 2010 |
| DENV-2 | Asian I | KY672948 | *Homo sapiens* | China | 2013 |
| DENV-2 | Asian I | LC410184 | *Homo sapiens* | Thailand | 2016 |
| DENV-2 | Asian I | LC410188 | *Homo sapiens* | Thailand | 2017 |
| DENV-2 | Asian I | KY672946 | *Homo sapiens* | China | 2015 |
| DENV-2 | Asian I | MN923120 | *Homo sapiens* | China | 2019 |
| DENV-2 | Asian I | JF968033 | *Homo sapiens* | Thailand | 2010 |
| DENV-2 | Asian I | MG895031 | *Homo sapiens* | Thailand | 2013 |
| DENV-2 | Asian I | JN415494 | *Homo sapiens* | Indonesia | 2010 |
| DENV-2 | Asian I | KR919813 | *Homo sapiens* | Indonesia | 2012 |
| DENV-2 | Asian I | KR919815 | *Homo sapiens* | Papua New Guinea | 2011 |
| DENV-2 | Asian I | KR919814 | *Homo sapiens* | Papua New Guinea | 2014 |
| DENV-2 | Asian I | DQ285553 | *Homo sapiens* | Reunion | 2004 |
| DENV-2 | Asian I | JN415513 | *Homo sapiens* | Malaysia | 2010 |
| DENV-2 | Asian I | JX298570 | *Homo sapiens* | Fiji | 2011 |
| DENV-2 | Asian I | KM279391 | *Homo sapiens* | Fiji | 2014 |
| DENV-2 | Asian I | KX357994 | *Homo sapiens* | Myanmar | 2015 |
| DENV-2 | Asian I | MK780872 | *Homo sapiens* | Thailand | 2015 |
| DENV-2 | Asian I | KY038915 | *Homo sapiens* | China | 2014 |
| DENV-2 | Asian I | MK780874 | *Homo sapiens* | Thailand | 2016 |
| DENV-2 | Asian I | LC410188 | *Homo sapiens* | Thailand | 2017 |
| DENV-2 | Asian I | LC410184 | *Homo sapiens* | Thailand | 2016 |
| DENV-2 | Asian I | MK780871 | *Homo sapiens* | Thailand | 2015 |
| DENV-2 | Asian I | KX262927 | *Homo sapiens* | China | 2015 |
| DENV-2 | Asian I | KX262943 | *Homo sapiens* | China | 2015 |
| DENV-2 | Asian I | KJ470760 | *Homo sapiens* | Myanmar | 2013 |
| DENV-2 | Asian I | KU509273 | *Homo sapiens* | Thailand | 2011 |
| DENV-2 | Asian I | KY851468 | *Homo sapiens* | Thailand | 2012 |
| DENV-2 | Asian I | JN568282 | *Homo sapiens* | Vietnam | 2010 |
| DENV-2 | Asian I | JF967986 | *Homo sapiens* | Cambodia | 2009 |
| DENV-2 | Asian I | JN568244 | *Homo sapiens* | Laos | 2010 |
| DENV-2 | Asian I | JN568274 | *Homo sapiens* | Thailand | 2010 |
| DENV-2 | Asian I | GU131932 | *Homo sapiens* | Cambodia | 2008 |
| DENV-2 | Asian I | LC410185 | *Homo sapiens* | Thailand | 2016 |
| DENV-2 | Asian I | KT175135 | *Homo sapiens* | Thailand | 2014 |
| DENV-2 | Asian I | KY851466 | *Homo sapiens* | Thailand | 2013 |
| DENV-2 | Asian I | JN568273 | *Homo sapiens* | Thailand | 2007 |
| DENV-2 | Asian I | LC147056 | *Homo sapiens* | Laos | 2013 |
| DENV-2 | Asian I | JF812111 | *Homo sapiens* | Thailand | 2006 |
| DENV-2 | Asian I | FJ898452 | *Homo sapiens* | Thailand | 2003 |
| DENV-2 | Asian I | EU482784 | *Homo sapiens* | Vietnam | 2007 |
| DENV-2 | Asian I | KT781555 | *Homo sapiens* | Australia | 2013 |
| DENV-2 | Asian I | KT175111 | *Homo sapiens* | Taiwan | 2011 |
| DENV-2 | Asian I | FJ639704 | *Homo sapiens* | Cambodia | 2003 |
| DENV-2 | Asian I | GQ868542 | *Homo sapiens* | Thailand | 1994 |
| DENV-2 | Asian I | DQ181798 | *Homo sapiens* | Thailand | 1999 |
| DENV-2 | Asian I | NC001474 | *Homo sapiens* | Thailand | 1964 |
| DENV-2 | Asian II | KX901652 | *Homo sapiens* | Cambodia | 2015 |
| DENV-2 | Asian II | GU211755 | *Homo sapiens* | Vietnam | 2006 |
| DENV-2 | American/Asian | JF730050 | *Homo sapiens* | USA | 2007 |
| DENV-2 | American/Asian | HQ891024 | *Homo sapiens* | Taiwan | 2008 |
| DENV-2 | American | GQ868592 | *Homo sapiens* | Colombia | 1986 |
| DENV-2 | American | HM582099 | *Homo sapiens* | Fiji | 1971 |
| DENV-2 | Cosmopolitan | KU365903 | *Homo sapiens* | Taiwan | 2015 |
| DENV-2 | Cosmopolitan | MN018349 | *Homo sapiens* | China | 2015 |
| DENV-2 | Cosmopolitan | MT252649 | *Homo sapiens* | Singapore | 2016 |
| DENV-2 | Cosmopolitan | KT781568 | *Homo sapiens* | Indonesia | 2015 |
| DENV-2 | Cosmopolitan | MK564477 | *Homo sapiens* | China | 2016 |
| DENV-2 | Cosmopolitan | KT781537 | *Homo sapiens* | Thailand | 2012 |
| DENV-2 | Cosmopolitan | MF004385 | *Homo sapiens* | France | 2014 |
| DENV-2 | Cosmopolitan | KC762671 | *Homo sapiens* | Indonesia | 2008 |
| DENV-2 | Cosmopolitan | KX380818 | *Homo sapiens* | Singapore | 2012 |
| DENV-2 | Cosmopolitan | KU517847 | *Homo sapiens* | Philippines | 2015 |
| DENV-2 | Cosmopolitan | LC436669 | *Homo sapiens* | Bangladesh | 2017 |
| DENV-2 | Cosmopolitan | MT180479 | *Homo sapiens* | Sri Lanka | 2017 |
| DENV-2 | Cosmopolitan | MT006184 | *Homo sapiens* | Sri Lanka | 2018 |
| DENV-2 | Cosmopolitan | MK513444 | *Homo sapiens* | Singapore | 2015 |
| DENV-2 | Cosmopolitan | MK629884 | *Homo sapiens* | South Korea | 2015 |
| DENV-2 | Cosmopolitan | MF314189 | *Homo sapiens* | Singapore | 2016 |
| DENV-2 | Cosmopolitan | MH488959 | *Homo sapiens* | Malaysia | 2014 |
| DENV-2 | Cosmopolitan | KX372564 | *Homo sapiens* | Australia | 2015 |
| DENV-2 | Cosmopolitan | KX452017 | *Homo sapiens* | Malaysia | 2014 |
| DENV-2 | Cosmopolitan | KX621246 | *Homo sapiens* | China | 2015 |
| DENV-2 | Cosmopolitan | MH827541 | *Homo sapiens* | China | 2017 |
| DENV-2 | Cosmopolitan | MK564479 | *Homo sapiens* | China | 2016 |
| DENV-2 | Cosmopolitan | MN923117 | *Homo sapiens* | China | 2019 |
| DENV-2 | Cosmopolitan | LC410190 | *Homo sapiens* | Thailand | 2016 |
| DENV-2 | Cosmopolitan | LC410189 | *Homo sapiens* | Thailand | 2016 |
| DENV-2 | Cosmopolitan | LC410191 | *Homo sapiens* | Thailand | 2017 |
| DENV-4 | I | MG895372 | *Homo sapiens* | Taiwan | 2015 |
| DENV-4 | I | LC410203 | *Homo sapiens* | Thailand | 2017 |
| DENV-4 | I | LC410202 | *Homo sapiens* | Thailand | 2017 |
| DENV-4 | I | MK780893 | *Homo sapiens* | Thailand | 2016 |
| DENV-4 | I | MK629501 | *Homo sapiens* | Indonesia | 2015 |
| DENV-4 | I | KY427081 | *Homo sapiens* | Indonesia | 2016 |
| DENV-4 | I | MK780889 | *Homo sapiens* | Thailand | 2015 |
| DENV-4 | I | MG601754 | *Homo sapiens* | China | 2013 |
| DENV-4 | I | MK780892 | *Homo sapiens* | Thailand | 2016 |
| DENV-4 | I | MG564136 | *Homo sapiens* | Thailand | 2015 |
| DENV-4 | I | MG895331 | *Homo sapiens* | Taiwan | 2013 |
| DENV-4 | I | MN955687 | *Homo sapiens* | Thailand | 2018 |
| DENV-4 | I | MG895369 | *Homo sapiens* | Taiwan | 2015 |
| DENV-4 | I | LC410200 | *Homo sapiens* | Thailand | 2016 |
| DENV-4 | I | LC410199 | *Homo sapiens* | Thailand | 2016 |
| DENV-4 | I | MK780897 | *Homo sapiens* | Thailand | 2016 |
| DENV-4 | I | MG895384 | *Homo sapiens* | Taiwan | 2015 |
| DENV-4 | I | KU509296 | *Homo sapiens* | Thailand | 2013 |
| DENV-4 | I | KU509300 | *Homo sapiens* | Thailand | 2013 |
| DENV-4 | I | KX357898 | *Homo sapiens* | Myanmar | 2015 |
| DENV-4 | I | MN955691 | *Homo sapiens* | Thailand | 2018 |
| DENV-4 | I | MH893694 | *Homo sapiens* | Thailand | 2016 |
| DENV-4 | I | KX224312 | *Homo sapiens* | Singapore | 2014 |
| DENV-4 | I | MN018397 | *Homo sapiens* | China | 2016 |
| DENV-4 | I | MG840524 | *Homo sapiens* | China | 2015 |
| DENV-4 | I | KJ470765 | *Homo sapiens* | Myanmar | 2013 |
| DENV-4 | I | MN955693 | *Homo sapiens* | Thailand | 2018 |
| DENV-4 | I | MK614088 | *Homo sapiens* | China | 2019 |
| DENV-4 | I | MH893695 | *Homo sapiens* | Myanmar | 2016 |
| DENV-4 | I | KT750007 | *Homo sapiens* | Thailand | 2013 |
| DENV-4 | I | MG895333 | *Homo sapiens* | Taiwan | 2013 |
| DENV-4 | I | MG895351 | *Homo sapiens* | Taiwan | 2014 |
| DENV-4 | I | MH893690 | *Homo sapiens* | China | 2013 |
| DENV-4 | I | MG895366 | *Homo sapiens* | Taiwan | 2015 |
| DENV-4 | I | KJ470764 | *Homo sapiens* | Myanmar | 2013 |
| DENV-4 | I | KT452793 | *Homo sapiens* | Myanmar | 2008 |
| DENV-4 | I | JF967792 | *Homo sapiens* | Myanmar | 2010 |
| DENV-4 | IIA | HQ332176 | *Homo sapiens* | Brazil | 2013 |
| DENV-4 | IIA | KP332176 | *Homo sapiens* | Venezuela | 2007 |
| DENV-4 | IIB | KU529756 | *Homo sapiens* | Indonesia | 2014 |
