# Supplementary material for: Dengue virus in humans and mosquitoes and their molecular characteristics in northeastern Thailand 2016-2018
Source: PLoS One. 2021 Sep 14;16(9):e0257460. doi: 10.1371/journal.pone.0257460 (PMC8439490; doi:10.1371/journal.pone.0257460)
Supplement: S3 Table — The molecular characteristics of 24 pairs of DENV positive samples were shown in this table. Gray row label represents serotype matched between patients and mosquitoes from their resident area. (DOCX) [file pone.0257460.s004.docx]

**S3 Table. Molecular characteristic of dengue virus between patient and adult mosquitoes collected from patient’s resident area.** The molecular characteristics of 24 pairs of DENV positive samples were shown in this table. Gray row label represents serotype matched between patients and mosquitoes from their resident area.

| **Patient ID** | **Year of collection** | **DENV serotype** | **DENV genotype** | | **Mosquito ID** | | **No. of mosquito per pooled** | | **DENV serotype** | | **DENV genotype** | | |
| --- | --- | --- | --- | --- | --- | --- | --- | --- | --- | --- | --- | --- | --- |
| 4005-0032-0 | 2016 | 1 | | NT | | 4005-0032 | | 1 | | 1,3 | | NT |  |
| 4010-0002-0 | 2016 | 1 | | I | | 4010-0002 | | 7 | | 4 | | NT |  |
| 4017-0014-0 | 2017 | 1 | | NT | | 4017-0014 | | 1 | | 2,3 | | NT |  |
| 4024-0002-0 | 2016 | 1 | | I | | 4024-0002 | | 2 | | 3 | | NT |  |
| 4024-0007-0 | 2016 | 1,4 | | (1) I | | 4024-0007 | | 13 | | 1,3 | | (1) I |  |
| 4405-0037-0 | 2018 | 1 | | NT | | 4405-0037 | | 2 | | 3 | | NT |  |
| 4505-0003-0 | 2018 | 1 | | NT | | 4505-0003 | | 10 | | 1 | | NT |  |
| 4507-0017-0 | 2016 | 1,4 | | (1) I | | 4507-0017 | | 2 | | 4 | | NT |  |
| 4017-0008-0 | 2016 | 2,4 | | NT | | 4017-0008 | | 13 | | 3 | | NT |  |
| 4405-0036-0 | 2018 | 2,3 | | NT | | 4405-0036 | | 2 | | 2,3 | | NT |  |
| 4603-0005-0 | 2018 | 2 | | NT | | 4603-0005 | | 22 | | 1,2,3 | | NT |  |
| 9918-0002-0 | 2018 | 2 | | Cosmopolitan | | 9918-0002 | | 6 | | 2 | | NT |  |
| 9918-0008-0 | 2018 | 2 | | NT | | 9918-0008 | | 3 | | 2,3 | | NT |  |
| 4005-0006-0 | 2016 | 3,4 | | (4) I | | 4005-0006 | | 5 | | 2,3,4 | | NT |  |
| 4005-0021-0 | 2016 | 3,4 | | NT | | 4005-0021 | | 6 | | 2,3 | | NT |  |
| 4024-0008-0 | 2016 | 3 | | NT | | 4024-0008 | | 10 | | 1 | | I |  |
| 4603-0025-0 | 2018 | 3 | | NT | | 4603-0029 | | 2 | | 3 | | NT |  |
| 4603-0029-0 | 2018 | 3 | | NT | | 4603-0029 | | 5 | | 2,3 | | NT |  |
| 9901-0007-0 | 2018 | 3 | | NT | | 9901-0007 | | 1 | | 2,3 | | NT |  |
| 4005-0005-0 | 2016 | 4 | | NT | | 4005-0005 | | 11 | | 1,2,3 | | NT |  |
| 4005-0007-0 | 2016 | 4 | | NT | | 4005-0007 | | 8 | | 4 | | I |  |
| 4017-0011-0 | 2016 | 4 | | NT | | 4017-0011 | | 11 | | 3 | | NT |  |
| 4507-0018-0 | 2016 | 4 | | I | | 4507-0018 | | 2 | | 1,2,3 | | NT |  |
| 4605-0001-0 | 2017 | 4 | | I | | 4605-0001 | | 6 | | 3 | | NT |  |

*Dengue virus serotype was determined by RT-PCR; Dengue virus genotype was determined by phylogenetic analysis base on envelope gene sequence; NT, not test; (1)I, DENV-1 genotype I; (4) I, DENV-4 genotype I; Cosmopolitan, genotype cosmopolitan.
