# Supplementary material for: Dengue virus in humans and mosquitoes and their molecular characteristics in northeastern Thailand 2016-2018
Source: PLoS One. 2021 Sep 14;16(9):e0257460. doi: 10.1371/journal.pone.0257460 (PMC8439490; doi:10.1371/journal.pone.0257460)
Supplement: S4 Table — (DOCX) [file pone.0257460.s005.docx]

**S4 Table. Age of 75 dengue patients who have mosquito inside the residence area.**

| **Growth stages** | **Age range** | **Number of cases** | **% Of cases** |
| --- | --- | --- | --- |
| Roughly equivalent to youth | 0-14 | 44 | 59 |
| Young adult | 15-24 | 22 | 29 |
| Middle adult | 25-44 | 6 | 8 |
| Old adult | 45-64 | 2 | 3 |
| Retirement | ≥65 | 1 | 1 |
| Total | | 75 | 100 |
